# Preparation and Performance of PVDF-HFP/PAN-Based Gel Polymer Electrolytes

**DOI:** 10.3390/gels11050317

**Published:** 2025-04-24

**Authors:** Xiubing Yao, Lingxiao Lan, Qiankun Hun, Xuanan Lu, Jianghua Wei, Xinghua Liang, Pengcheng Shen, Ying Long, Yifeng Guo

**Affiliations:** 1 Guangxi Key Laboratory of Automobile Components and Vehicle Technology, Guangxi University of Science & Technology, Liuzhou 545006, China; 18300084899@163.com (X.Y.); llx2685062@163.com (L.L.); 13383371921@163.com (Q.H.); a15007751851@163.com (X.L.); 18852066673@163.com (P.S.); 2 School of Information Science and Engineering, Liuzhou Institute of Technology, Liuzhou 545616, China; 18178806880@163.com; 3 School of Mechanical Engineering, Chengdu University, Chengdu 610106, China; guobujia2000@163.com

**Keywords:** UV curing process, blending, LLZTO, lithium-ion batteries

## Abstract

Solid-state electrolytes are widely expected to enhance the performance of lithium-ion batteries, providing higher energy density and improved safety. However, challenges still need to be solved in their practical application due to low ionic conductivity and high interfacial resistance at room temperature. In this study, we successfully developed a high-performance gel polymer electrolyte (GPEs) by blending poly(vinylidene fluoride-co-hexafluoropropylene)(PVDF-HFP) and polyacrylonitrile (PAN) through UV curing, cross-linking with ethoxylated trimethylolpropane triacrylate (ETPTA), and incorporating Li_6.4_La_3_Zr_1.4_Ta_0.6_O_12_ (LLZTO). At room temperature, the ionic conductivity of the GPEs was 2.8 × 10^−4^ S/cm, with a lithium-ion transference number of 0.6. Moreover, during lithium plating/stripping tests, the assembled Li/PPEL/Li symmetric cell exhibited stable cycling for up to 600 h at a current density of 0.1 mA/cm^2^. Notably, the GPEs enabled the LiFePO_4_/GPEs/Li battery to achieve excellent performance, delivering high discharge capacities at room temperature (164.3 mAh g^−1^ at 0.1 C and 88.8 mAh g^−1^ at 1 C), with a capacity retention of 89.4% after 200 cycles at 0.5 C. Therefore, solid-state batteries using this electrolyte exhibit excellent performance, including adequate capacity and cycling stability.

## 1. Introduction

With the rapid development of portable electronic devices and smart grids, the demand for higher energy density and safety has become increasingly critical. Lithium metal anodes, with their lowest reduction potential and highest theoretical specific capacity, have become a key component in advancing lithium-ion batteries [1,2,3]. Meanwhile, the rapid emergence of flexible and wearable electronics has imposed unprecedented requirements on energy storage systems, particularly in terms of mechanical adaptability and safety under dynamic deformation [4,5,6,7,8]. Solid-state batteries, which use lithium metal as the anode, offer significant advantages in higher energy density and improved safety, making them a growing focus in energy storage systems [9,10,11]. However, traditional lithium-ion batteries are liquid-based, and their liquid electrolytes present severe toxicity and poor electrochemical stability, which pose significant safety risks and hinder their commercialization [12,13,14]. Replacing conventional organic electrolytes with gel polymer electrolytes (GPEs) has emerged as an effective strategy to address these safety concerns, garnering widespread attention and extensive research [15,16]. The development of gel polymer electrolytes (GPEs) is increasingly focused on sustainability and scalability, with bio-based materials gaining traction [17,18]. Biopolymer-based GPEs show promise in applications like Li-ion batteries by suppressing side reactions and dendrite growth. Yet, these emerging sustainable GPEs generally lag behind established formulations in performance. Balancing eco-friendliness with maintaining high performance remains a key challenge as the field of GPEs advances [19,20,21].

Polymer solid-state electrolytes (SPEs) offer advantages, such as flexibility and ease of processing, allowing for good contact with both the anode and cathode, which helps reduce interfacial resistance [16]. However, SPEs also have notable drawbacks, including high crystallinity that limits ion conductivity and weak molecular interactions that make them unstable at high voltages [22]. GPEs combine the advantages of liquid and solid electrolytes, provide a promising solution [23,24]. These materials exhibit higher ionic conductivity and excellent mechanical properties, effectively suppressing lithium dendrite growth and enhancing the optimized contact at the electrode interfaces, thus opening new avenues for advancing solid-state electrolytes [25,26,27]. Poly(vinylidene fluoride-co-hexafluoropropylene)(PVDF-HFP), a polymer electrolyte with a partially amorphous and partially crystalline structure, has a relatively high ionic conductivity and low crystallinity compared to other polymers [28,29,30]. Yadav et al. successfully incorporated LLZTO ceramic particles into PVDF-HFP/LiTFSI to prepare a composite solid polymer electrolyte with an ionic conductivity of 1.04 × 10^−4^ S/cm and a lithium-ion transference number of 0.26, with an electrochemical window of 4.7 V. Furthermore, a solid-state LiFePO_4_/CSPE-20/Li battery delivered a high first discharge capacity of 131 mAh g^−1^. The as-prepared Li||0.2PESF-0.8LLZTO CPEs||LiFePO_4_ full cells deliver an initial discharge capacity of 159.8 mAh g^−1^ at 0.1 C and successfully operated for over 80 cycles [31]. With its high antioxidant capability, polyacrylonitrile(PAN) is well-suited for use with high-voltage cathode materials, offering high ionic conductivity, thermal stability, and compatibility with lithium electrodes. Among the inorganic fillers used in polymers, Li_6.4_La_3_Zr_1.4_Ta_0.6_O_12_(LLZTO) has emerged as the most thoroughly studied filler, with an ionic conductivity as high as 10^−3^ S cm^−1^ [32,33]. Gao et al. show a large-scale feasible method to prepare a succinonitrile(SN)/PAN coated Li_6.4_La_3_Zr_1.4_Ta_0.6_O_12_ (LLZTO) with flexibility and high ionic conductivity by tape-casting. At room temperature, it has an ion conductivity of 4 × 10^−4^ S/cm, a lithium-ion transference number of 0.65, and an electrochemical window of 4.8 V. In this way, a long lifespan of over 500 cycles and a high discharge capacity (163 mAh g^−1^) are achieved based on LiFePO4 (LFP) cathodes at 0.2 C [34]. Additionally, LLZTO powder helps reduce crystallinity, thereby improving the migration efficiency of lithium ions in the electrolyte [35,36].

In this study, we modified PVDF-HFP-based gel polymer electrolytes using a blending technique supplemented with inorganic active fillers. A novel porous flexible GPEs was successfully prepared using UV curing. The membrane was constructed with a PVDF-HFP and PAN blend as the matrix, with ethoxylated trimethylolpropane triacrylate (ETPTA) added for cross-linking and LLZTO incorporated as the inorganic active filler. This approach holds great promise for application in next-generation high-performance energy storage systems.

## 2. Results and Discussion

**Figure 1:**
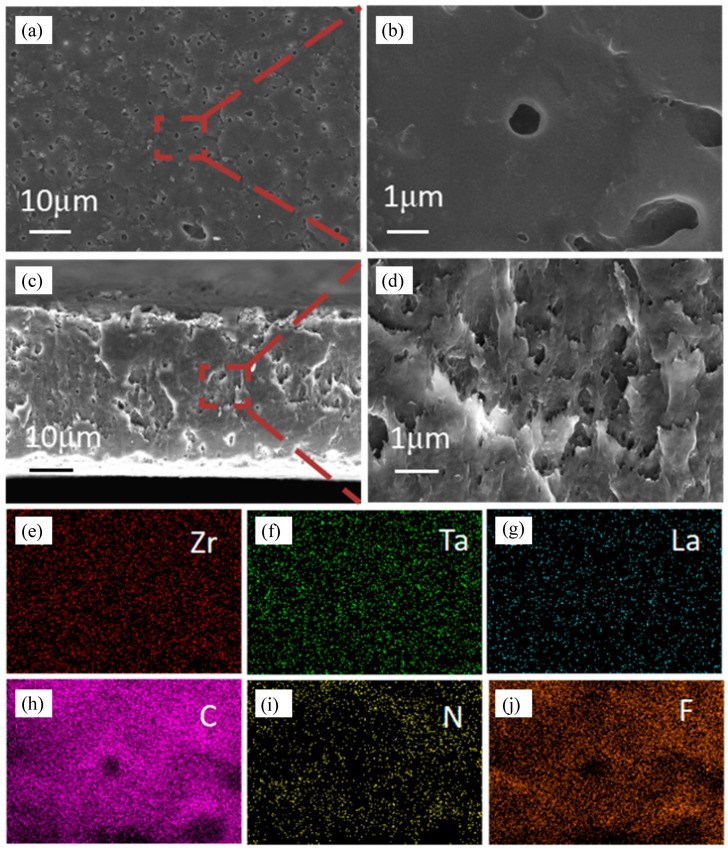
SEM map of solid electrolyte (**a**,**b**) PPEL surface; (**c**,**d**) SEM map of PPEL cross-section; (**e**–**j**) EDS element mapping; analyzed the distribution of Zr, Ta, La, C, N, F elements in the sample electrolyte.

Figure 1a,b shows the surface SEM images and the magnified view of the PPEL. The surface of the solid-state electrolyte exhibits uniformly distributed micro-sized pores rich in voids. This is attributed to the evaporation of the organic solvent during the drying process, which leads to the formation of evenly distributed channels on the electrolyte surface. These micropores provide additional pathways for lithium-ion transport, enhancing electrochemical performance. Additionally, the smooth surface of the electrolyte membrane facilitates better interface contact between the electrode and the electrolyte [37,38]. It has been reported that porous structures are a promising approach to redistributing lithium-ion flux, enabling more uniform lithium deposition [39]. Figure 1c,d shows the cross-sectional SEM images and magnified views of the PPEL. The cross-section reveals abundant interconnected pores, which are beneficial for enhancing ionic conductivity. EDS analysis of the GPEs was performed to examine the distribution of LLZTO in the electrolyte. Figure 1e–j presents the surface EDS spectra for six elements: Zr, Ta, La, C, N, and F. The distribution of these elements confirms that the inorganic filler LLZTO is uniformly dispersed within the polymer matrix.

**Figure 2:**
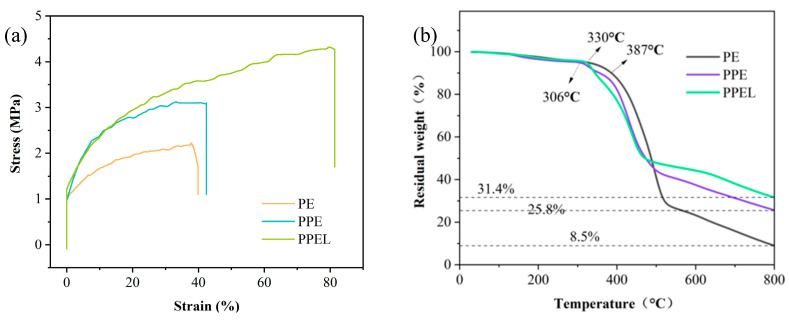
(**a**) Stress–strain curves of PE, PPE, and PPEL membranes; (**b**) thermogravimetric analysis of GPEs of PE, PPE, and PPEL.

Lithium-ion batteries’ mechanical stability largely depends on the strength of the solid-state electrolyte [40]. When the internal stress exceeds the strength of the solid electrolyte, mechanical failure occurs, negatively impacting the battery’s electrochemical performance. As shown in Figure 2a, the tensile strength of PE is 2.21 MPa with an elongation of 39%; the tensile strength of PPE is 3.1 MPa with an elongation of 42.4%; and the tensile strength of PPEL is 4.3 MPa with an elongation of 81.4%. LLZTO exhibits a high modulus, high aspect ratio, and strong interfacial bonding with the matrix, facilitating multi-directional fracture and tensile stress sharing, which is critical for suppressing lithium dendrite growth during cycling [41].

We also performed thermogravimetric analysis (TGA) to assess the thermal stability of the solid-state electrolyte membranes, with the results shown in Figure 2b. The weight loss observed at temperatures below 180 °C is attributed to the GPEs’ absorption of moisture from the air. After 180 °C, a slight weight loss is due to the residual DMF solvent in the solid electrolyte. PE decomposes at around 390 °C, PPE at around 310 °C, and PPEL at around 330 °C. At a decomposition temperature of 800 °C, the mass loss rates for PE, PPE, and PPEL are 91.5%, 74.2%, and 68.6%, respectively. By comparing the decomposition temperatures, it can be inferred that the lower decomposition temperature of PPE, compared to PE, is due to the lower decomposition temperature of PAN (around 250 °C) when blended with PVDF-HFP. Comparing the weight loss rates shows that PPEL has the least mass loss, indicating that the incorporation of LLZTO improves the thermal stability of the GPEs. With a high decomposition temperature of around 300 °C, the GPEs significantly exceed the typical operating temperature range of lithium batteries in commercial applications, demonstrating that their performance meets the requirements for lithium-ion battery commercialization.

**Figure 3:**
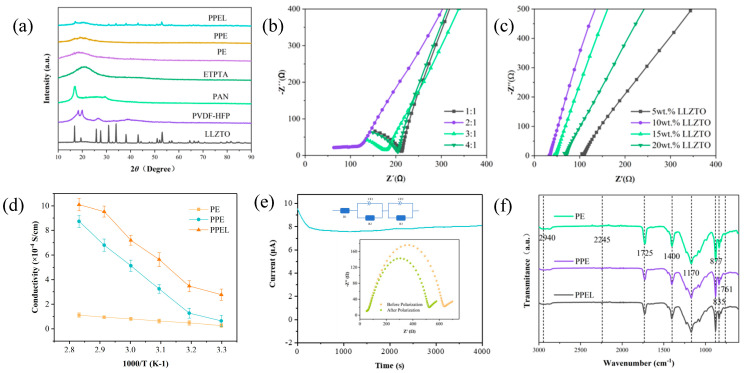
(**a**) XRD patterns of GPEs. EIS impedance diagram of solid electrolyte at room temperature (**b**) PPE; (**c**) PPEL; (**d**) Ionic conductivity values of PE, PPE, and PPEL at different temperatures; (**e**) DC polarization curves of the Li/PPEL/Li batteries at a polarization voltage of 10 mV; (**f**) PE, PPE and PPEL infrared spectrum characterization diagram.

Figure 3a shows the XRD patterns of LLZTO powder, PVDF-HFP, PAN, ETPTA, and composite solid-state electrolytes (PE, PPE, and PPEL). The results indicate that PVDF-HFP exhibits a semi-crystalline structure, with three diffraction peaks at approximately 18.8°, 20.5°, and 39.2°. After UV irradiation, the characteristic peaks of PVDF-HFP almost disappear, and the overall peak around 2θ = 19° becomes broadened. This suggests that the ETPTA monomer has polymerized within the PVDF-HFP, reducing its crystallinity. When PAN is added to PE, a peak similar to the PE peak but with lower intensity remains, indicating that adding PAN further reduces the crystallinity of the solid-state electrolyte. After the introduction of LLZTO, the overall peak intensity of PPEL is noticeably lower than that of PE and PPE, demonstrating that the inorganic filler LLZTO reduces the crystallinity of the blended polymer, leading to a more disordered state of the polymer chains. Compared to LLZTO powder, the characteristic peak positions of LLZTO in the PPEL membrane do not show significant changes, indicating that LLZTO powder is stably integrated into the PVDF-HFP/PAN matrix.

Ion conductivity is a crucial indicator of GPEs. Figure 3b,c shows the Nyquist plots for the PPE and PPEL samples. Figure 3b shows that when the PVDF-HFP: PAN ratio is 2:1, the bulk impedance is minimized, and the ion conductivity reaches 6.1 × 10^−5^ S/cm. The introduction of LLZTO reduces the polymer crystallinity, expands the amorphous region [42], and creates new pathways for lithium-ion transport, allowing more Li^+^ to move freely within the solid-state electrolyte. Figure 3c presents the ion conductivity of PVDF-HFP/PAN-based solid-state electrolytes with varying amounts of LLZTO (5 wt.%, 10 wt.%, 15 wt.%, and 20 wt.%). The highest ion conductivity of 2.8 × 10^−4^ S/cm is achieved when LLZTO content is 10 wt.%. However, as LLZTO content increases beyond this point, ion conductivity begins to decrease due to the agglomeration of excess LLZTO, which hinders Li^+^ transport and reduces the overall conductivity [43]. Figure 3d shows the ion conductivity values of PE, PPE, and PPEL at different temperatures, confirming that the highest ion conductivity is achieved with 10% LLZTO. LLZTO doped with Ta exhibits a cubic phase [44], which can provide more ion channels and result in higher ion conductivity.

The lithium-ion transference number reflects the effective transference of lithium ions in GPEs. A high lithium-ion transference number contributes to anion transference, reducing concentration polarization [45]. Figure 3e shows the DC polarization curves and the electrochemical impedance spectroscopy (EIS) data before and after polarization for the symmetric Li/PPEL/Li cell. The lithium-ion transference number of polymer solid-state electrolytes (SPEs) typically ranges from 0.2 to 0.4 [46]. In contrast, the lithium-ion transference number of PPEL reaches 0.6, significantly higher than traditional SPEs. This improvement is attributed to the abundant F-groups in PVDF-HFP, which interact with lithium ions and allow for faster ion transport, thus enhancing the lithium-ion transference number of the solid-state electrolyte membrane and promoting lithium-ion migration. The significant improvement can also be ascribed to the LLZTO filler, an active lithium-ion conductor with a transference number of 1 [47]. Therefore, the incorporation of LLZTO powder as an inorganic filler not only increases ion conductivity but also has a positive impact on the lithium-ion transference number. In addition, the –C≡N groups in PAN can interact with free Li^+^ ions, which also contribute to the enhancement of the lithium-ion transference number [48].

Fourier-transform infrared spectroscopy (FTIR) was used to confirm the quantitative changes in the bands of the blended polymer (Figure 3f). The FTIR spectra reveal the chemical interactions in the three GPEs: PE, PPE, and PPEL. The peaks at 761 cm^−1^ and 877 cm^−1^ correspond to the α-phase of PVDF-HFP, while the peaks at 836 cm^−1^ and 1170 cm^−1^ are associated with the -CF_2_ stretching vibration, and the peak at 1400 cm^−1^ corresponds to C-F stretching. These results confirm that PVDF-HFP is a semi-crystalline copolymer. After blending with PAN, the intensity of the characteristic PVDF-HFP peaks weakens, indicating polymer interactions within the PPE. Additionally, two peaks at 2940 cm^−1^ and 2245 cm^−1^ in the PPE spectrum are attributed to the symmetric -CH_2_ and -CN stretching vibrations of the PAN polymer, respectively, confirming the successful preparation of the PVDF-HFP/PAN binary composite membrane. Upon introducing LLZTO powder into the PPE, the resulting PPEL electrolyte membrane shows similar spectral features, with no new or disappearing peaks observed. This suggests that the incorporation of nanoparticles into the polymer matrix results in simple physical property modification without inducing significant chemical changes.

**Figure 4:**
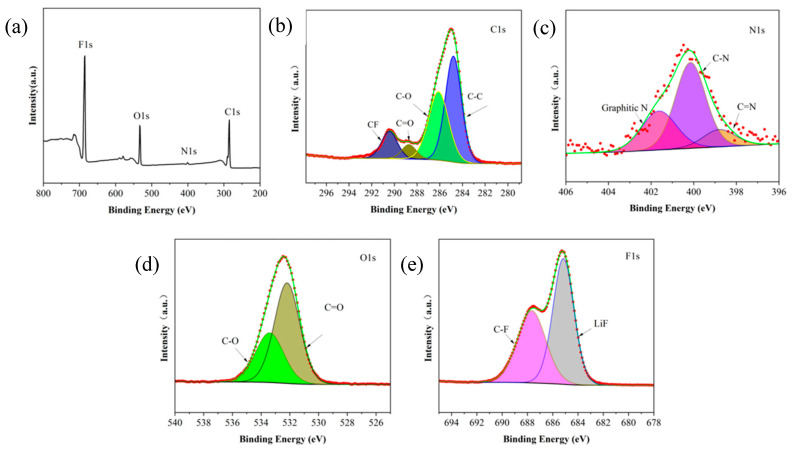
XPS spectra of PPEL GPEs after electrolyte membrane recycling. (**a**) Full spectrum diagram; (**b**) C1s; (**c**) C1s; (**d**) O1s; (**e**) N1s.

The X-ray photoelectron spectroscopy (XPS) spectrum of the cycled PPEL electrolyte is shown in Figure 4a. In the C1s spectrum (Figure 4b), four peaks are observed at 284.78 eV, 286.20 eV, 288.68 eV, and 290.52 eV, corresponding to C-C, C-O/C-N, C=O, and C-F bonds, respectively. The N1s spectrum (Figure 4c) can be fitted into three peaks, with characteristic peaks at 398.80 eV and 399.97 eV assigned to C=N and C-N bonds, respectively. Additionally, the peak at 401.63 eV is attributed to the nitrile group (-C≡N) from PAN. Similarly, in the O1s spectrum (Figure 4d), a strong peak at 532.19 eV and a weaker peak at 533.41 eV are attributed to C=O and C-O bonds, respectively. These oxygen species primarily originate from the LLZTO ceramic fillers incorporated in the polymer matrix [49]. Figure 4e shows the F1s peak at 685.1 eV, which reveals a high content of LiF (56.2%) in the generated solid electrolyte interphase (SEI) layer. A significant amount of LiF plays a crucial role in the passivation of the lithium metal surface and in delaying dendrite growth. The formation of the passivation layer helps protect the lithium metal surface and prevents further reactions with the electrolyte, thereby extending the battery’s cycle life. It has been reported that a high LiF content promotes the formation of the SEI layer, and LiF can effectively increase the mechanical strength of the SEI, suppressing the growth of lithium dendrites. Moreover, the LiF layer exhibits high interfacial energy and low surface diffusion barriers for Li-ions, allowing rapid lithium-ion transport to the lithium anode during deposition. As an excellent electronic insulator, LiF inhibits the transmission of electrons across the interface, effectively preventing electrolyte decomposition [50].

**Figure 5:**
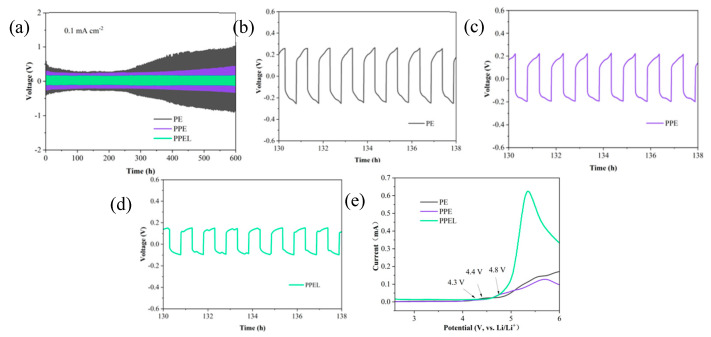
(**a**) Cycling performance of plating / lithium stripping at Li/PE, PE/PE, and PPEL/Li at 0.1 mA/cm^2^ current density; polarization voltage at 130–138 h (**b**) PPE; (**c**) PPE; (**d**) PPEL; (**e**) LSV profiles of GPEs membranes.

The long-term interface stability between the lithium metal anode and the solid-state electrolyte is critical for suppressing the formation and growth of lithium dendrites during lithium plating/stripping cycles [51]. To investigate the compatibility and dynamic stability of the PPEL GPEs with lithium metal, constant current stripping/plating tests were conducted on symmetric cells of Li/PE/Li, Li/PPE/Li, and Li/PPEL/Li, with the results shown in Figure 5a. For the Li/PE/Li symmetric cell, the polarization voltage initially reached 0.58 V. However, it decreased afterward due to the reaction of a small amount of electrolyte added at the beginning, which reacted with the electrodes. The polarization voltage stabilized at 0.26 V between 100 and 250 h (Figure 5b). However, after 250 h, the voltage started to rise, indicating the formation of lithium dendrites and an increase in interface resistance. In the Li/PPE/Li symmetric cell, the polarization voltage remained stable at 0.23 V for up to 350 h (Figure 5c), but with continued cycling, the overpotential rapidly increased. The poor mechanical properties of the GPEs (PE and PPE) led to uneven lithium plating and stripping, preventing stable polarization voltage. In contrast, the Li/PPEL/Li symmetric cell demonstrated a highly stable polarization voltage of 0.16 V over 600 h of cycling at a current density of 0.1 mA cm^−2^ (Figure 5d). This suggests a stable SEI (solid electrolyte interphase) layer formed between the electrode and electrolyte. Additionally, it can be concluded that adding LLZTO improves the interface stability between the solid-state electrolyte and the lithium metal anode. This is likely due to the LLZTO particles acting as a barrier, preventing reactions between the lithium anode and the polymer PVDF-HFP. These electrochemical performance results are highly competitive compared to previously reported GPEs designs.

In pursuing high stability and safety in lithium-ion battery technology, the electrochemical stability of GPEs plays a crucial role. As the voltage increases, electrolyte decomposition occurs. When a certain threshold is reached, intense redox reactions inside the battery are triggered, leading to a rapid increase in current. The results are shown in Figure 5e. For the PE electrolyte, the current only starts to increase gradually at 4.3 V. For PPE, the current begins to rise at 4.4 V, while for PPEL, the current sharply increases after 4.8 V. The sharp rise in current indicates the onset of oxidative decomposition of the electrolyte. This demonstrates that PPEL has a larger electrochemical window and superior electrochemical stability, making it highly promising for high-voltage lithium batteries.

**Figure 6:**
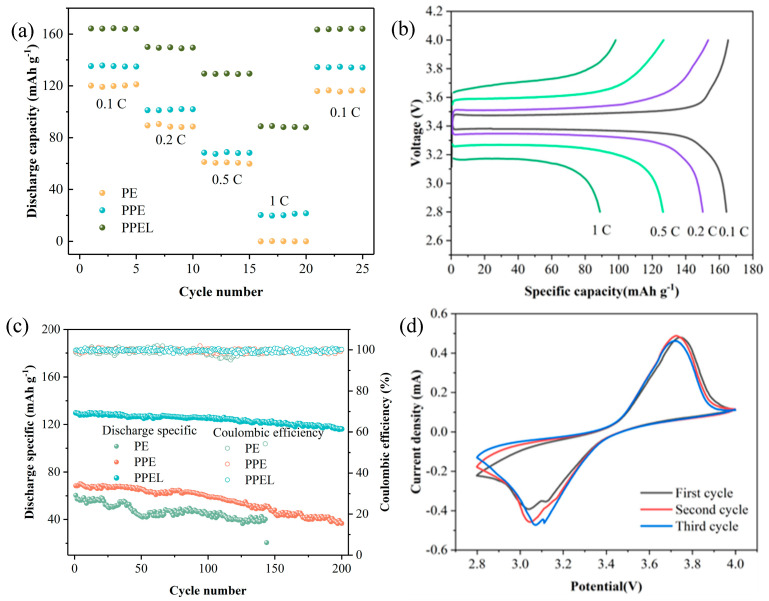
(**a**) The rate capability of PE-based LFP/Li, PPE 3:7-based LFP/Li, and PPEL-based LFP/Li batteries at 25 °C; (**b**) galvanostatic charge-discharge curves of PPEL-based LFP/Li batteries at 0.1, 0.2, 0.5, and 1 C; (**c**) cycling performances of PE-based LFP/Li, PPE-based LFP/Li, and PPEL-based LFP/Li batteries at 25 °C; (**d**) CV curves of lithium-ion batteries with PPEL electrolyte at a scan rate of 0.2 mV s^−^^1^.

To evaluate the practical feasibility of various GPEs, LFP/GPEs/Li coin batteries were assembled and tested at 25 °C. The rate performance of the batteries with three different electrolytes was studied over a current density range from 0.1 C to 1 C, as shown in Figure 6a. At 0.1 C, the PPEL battery delivered the highest discharge-specific capacity (164.3 mAh g^−1^). In contrast, the PE and PPE cells showed initial discharge capacities of 120.1 and 135.2 mAh g^−1^, respectively, with a difference of 44.2 and 29.1 mAh g^−1^ compared to PPEL. The capacity difference between the three solid-state electrolytes widened as the current density increased. The PPEL cell provided discharge capacities of 150.1, 129.4, and 88.8 mAh g^−1^ at 0.2 C, 0.5 C, and 1 C, respectively, while the PE cell showed capacities of 89.5 and 61.2 mAh g^−1^ at 0.2 C and 0.5 C, with no measurable capacity at 1 C. The PPE cell showed discharge capacities of 101.2, 68.5, and 20.3 mAh g^−1^ at the corresponding current densities. Upon returning to 0.1 C, the PPEL cell nearly regained its initial discharge capacity (163.5 mAh g^−1^). In contrast, the PE and PPE cells maintained 115.9 and 134.4 mAh g^−1^, representing 96.5% and 99.4% of their initial discharge capacity, respectively. The superior performance of PPEL over PE and PPE is attributed to the combined properties of PAN’s high absorptivity and the specific F-groups on the surface of PVDF-HFP, which facilitate lithium-ion transport. The charge–discharge curves of the PPEL cell at various current densities are shown in Figure 6b, where the observed low overpotentials indicate minimal polarization, which is beneficial for a long cycle life. These results demonstrate that the PVDF-HFP/PAN double polymer matrix significantly enhances Li^+^ transport, making it highly suitable for high-power-density energy storage systems. Additionally, the room temperature cycling performance of batteries with the three electrolytes was tested at a load of 2 mA cm^−2^ over 200 cycles at a current density of 0.5 C (Figure 6c). The initial discharge capacities were 60.4, 68.4, and 129.8 mAh g^−1^ for the PE, PPE, and PPEL cells, respectively. The PE cell short-circuited after 143 cycles, while the PPE cell retained a discharge capacity of 36.5 mAh g^−1^ after 200 cycles, with a capacity retention of 53.4%. In contrast, the PPEL cell maintained a discharge capacity of 116 mAh g^−1^ after 200 cycles, with a capacity retention of 89.4%. This indicates that the PPEL electrolyte provides superior long-term cycling stability due to the chemical/electrochemical equilibrium at the interface. Figure 6d shows the cyclic voltammetry (CV) curves of the LFP/PPEL/Li battery in the voltage range of 2.8 V–4.0 V (vs. Li/Li^+^) at a scan rate of 0.2 mV s^−1^. Two characteristic peaks were observed at 3.1 V and 3.7 V during the reduction and oxidation scans, respectively. The minimal change in the curves during cycling indicates that no significant polarization occurred, demonstrating that the PPEL-based battery exhibits high reversible charge–discharge behavior and excellent electrochemical stability. To better visualize the overall improvement, Table 1 provides a comparative summary of the key performance metrics for PE, PPE, and PPEL electrolytes.

## 3. Conclusions

In summary, we successfully prepared high-performance GPEs by blending PVDF-HFP and PAN via UV curing, cross-linking with ETPTA, and incorporating LLZTO. This design significantly enhances lithium-ion batteries’ safety, functionality, and electrochemical performance. At room temperature, the GPEs exhibits an ionic conductivity of 2.8 × 10^−4^ S/cm, a lithium-ion transference number of 0.6, an electrochemical window of 4.8 V, and a tensile strength of 4.3 MPa. Furthermore, in the lithium plating/stripping test, the assembled Li/PPEL/Li symmetrical cell demonstrated a stable cycling performance over 600 h at a current density of 0.1 mA cm^−2^. Notably, the GPEs enabled the LiFePO_4_/GPEs/Li lithium battery to achieve excellent performance, including high discharge capacities under high current densities (164.3 mAh g^−1^ at 0.1 C and 88.8 mAh g^−1^ at 1 C), and good cycling stability (capacity retention of 89.4% after 200 cycles at 0.5 C, with an average coulombic efficiency of 99.6%). Compared with other PVDF-HFP-based electrolyte, our system exhibits higher ionic conductivity and longer cycling stability, highlighting the advantage of our UV-curing and filler dispersion strategy. This work provides a practical approach for high-performance, safe solid-state lithium-ion batteries. Therefore, GPEs based on this design have the potential to exhibit promising electrochemical performance and contribute to the practical development of safe, high-energy lithium-ion batteries.

## 4. Materials and Methods

### 4.1. Preparation of Gel Polymer Electrolytes (GPEs)

Preparation of PE: 2 g of PVDF-HFP (Mn = 600,000, Arkema, Colombes, France) was dissolved in DMF solvent, and LiClO_4_ (99.99%, Aladdin Chemical Co., Shanghai, China) was added and thoroughly stirred. Then, an appropriate amount of ETPTA and HMPP was introduced, and the mixture was stirred for 5 min. The resulting precursor solution was poured onto a PTFE plate and subjected to UV curing. Finally, the cured membrane was placed in a drying oven for 6 h. The prepared GPE membrane is named PE and has a thickness of approximately 50 μm.

Preparation of PPE: 2 g of PVDF-HFP was dissolved in DMF solvent along with poly(acrylonitrile) (PAN, Mw = 85000, Shanghai, China) in varying ratios to PVDF-HFP (1:1, 2:1, 3:1, 4:1). After thorough mixing, LiClO_4_ was added. An appropriate amount of Plasticizers trimethylolpropane ethoxylate triacrylate (ETPTA, Mn = 428, Macklin, Shanghai, China) and 2-hydroxy-2-methylpropiophenone (HMPP, Cambridge, Beijing, China) was then introduced, and the mixture was stirred for 5 min. The precursor solution was poured onto a PTFE plate, subjected to UV curing, and dried in an oven for 6 h. The resulting GPEs film is named PEL and is approximately 50 μm thick.

**Figure 7:**
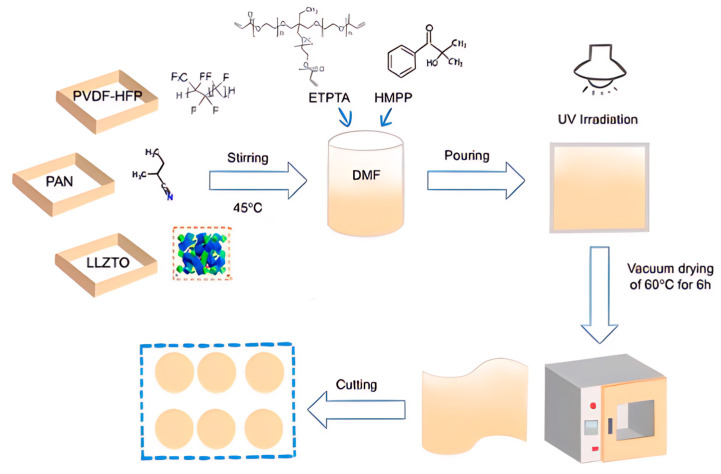
Preparation of PPEL GPEs membrane.

Preparation of PPEL: 2 g of PVDF-HFP and PAN (in a weight ratio 2:1) were dissolved in DMF solvent. After adding LiClO_4_ and mixing thoroughly, LLZTO was incorporated at different weight ratios (5 wt.%, 10 wt.%, 15 wt.%, 20 wt.) into the solution. An appropriate amount of ETPTA and HMPP was then added, stirring the mixture for 5 min. The precursor solution was poured onto a PTFE plate and subjected to UV curing. Finally, the membrane was dried in an oven for 6 h. The resulting GPE membrane is named PPEL and is approximately 50 μm thick, and a schematic of its preparation is shown in Figure 7.

### 4.2. Material Characterization

The cut Gel Polymer Electrolytes (GPEs) were characterized using X-ray diffraction (XRD, DX-2700, Dandong, China) with Cu-Kα radiation (40 kV × 30 mA). Surface and cross-sectional morphology of the GPEs was examined using scanning electron microscopy (SEM, Phenom Spectra G2, Shanghai, China). Cross-sectional samples were prepared by embrittling the GPEs with liquid nitrogen, followed by gold coating for imaging. The tensile strength of the rectangular GPEs samples was measured using a universal testing machine (WDW-5, Tenson, Jinan, China). Thermogravimetric analysis (TGA) was conducted under a nitrogen atmosphere using a Netzsch F3 Tarsus system (Beyern, Germany), with a temperature range of 30–800 °C and a heating rate of 10 °C/min. Fourier transform infrared spectroscopy (FTIR, Spectrum 100, PerkinElmer, Waltham, MA, USA) was used to identify the functional groups on the surface of the cut GPEs, within the wavenumber range of 400–4000 cm^−1^. X-ray photoelectron spectroscopy (XPS, Thermo Fisher Scientific K-Alpha+, Waltham, MA, USA) was employed to assess the bonding structure of the GPEs after cycling.

### 4.3. Electrochemical Properties

Electrochemical impedance spectroscopy (EIS) was performed at various temperatures, with an open-circuit voltage (OCV) ranging from 0.1 Hz to 1 MHz and an amplitude of 10 mV. GPEs were placed between two smooth stainless steel (SS) electrodes. Ionic conductivity (*σ*) of the electrolytes was determined from the EIS data using Equation (1):

(1) $$\sigma =\frac{L}{RS}$$

In this equation, *R* represents the bulk resistance measured by EIS, while *L* and *S* denote the thickness and effective area of the GPEs, respectively. The activation energy (*E_a_*) of each solid electrolyte is determined using the Arrhenius equation (Equation (2)):

(2) $$\sigma (T)=A\exp\left[-\frac{E_{a}}{RT}\right]$$

Here, *A* is the exponential prefactor, *E_a_* represents the activation energy associated with the ion skipping conduction process, and *T* is the absolute temperature. The electrochemical stability windows of different GPEs were assessed using linear scanning voltammetry (LSV) across a voltage range of 2 V to 6 V, the scan rate is 5 mV/s. Stainless steel (SS) was used as the working electrode and lithium metal as the counter/reference electrode. SS/ GPEs /Li assemblies were tested in CR2025 coin cells for LSV measurements. To evaluate the stability of various solid-state electrolytes in contact with the lithium anode, two polished lithium foils were used in a symmetrical cell configuration. To measure the lithium-ion transference number (*t_Li_^+^*), the same symmetrical cells containing different electrolytes were tested using the chronoamperometry method, with a 10 mV polarization applied for 4000 s. Simultaneously, the AC impedance spectra before and after polarization were recorded at an oscillation voltage of 10 mV, with a frequency range of 0.1 Hz to 1 MHz, and *t_Li_^+^* was calculated using Equation (3):

(3) $$t_{{Li}^{+}}=\frac{I_{s}\left(∆V-R_{0}I_{0}\right)}{I_{0}\left(∆V-R_{s}I_{s}\right)}$$

In this equation, *I*_0_ and *I*_S_ represent the initial and steady-state currents, respectively, *R*_0_ and *R*_S_ are the charge transfer resistances of the electrolyte system before and after polarization, and Δ*V* is the applied oscillation voltage of 10 mV. LFP/GPEs/Li configurations were assembled in CR2025 coin cells for Cyclic Voltammetry (CV) testing. The CV tests were performed at a scan rate of 0.2 mV/s.

Rate and cycle tests of the batteries were performed using a battery testing system (Neware, Dongguan, China). Commercial lithium iron phosphate (LFP) was used as the positive active material, and a lithium sheet served as the negative electrode. The positive electrode mixture consisted of LiFePO_4_ (LFP, ≥99.5%, Krud, Shanghai, China), PVDF, and conductive carbon (Super-P, ≥99.5%, Krud, Shanghai, China) in a weight ratio of 8:1:1. This mixture was dissolved in a solvent, and the resulting solution was applied to aluminum foil. The coated foil was then vacuum-dried for 36 h and cut into 14 mm discs for use in the tests. The mass loading was 2.4 mg/cm^2^. The electrochemical performance was evaluated within a voltage range of 2.8 to 4.0 V.

## Figures and Tables

**Table 1:** Performance comparison of PE, PPE, and PPEL.

GPEs	Mechanical Stability (MPa)	Ion Conductivity (S/cm)	Electrochemical Window (V)	Discharge Capacities (mAh/g)	Capacity Retention (0.2 C, 200 Cycles)
PE	2.16	9.1 × 10^−5^	4.3	120.1	short-circuited after 143 cycles
PPE	3.1	6.1 × 10^−5^	4.4	135.2	53.4%
PPEL	4.35	2.8 × 10^−4^	4.8	164.3	89.4%

## Data Availability

Data are contained within the article.
